# Supplementary material for: Splice-Junction-Based Mapping of Alternative Isoforms in the Human Proteome
Source: Cell Rep. Author manuscript; Available in PMC 2020 Jan 15. (PMC6961840; doi:10.1016/j.celrep.2019.11.026)
Supplement: 2 [file NIHMS1546469-supplement-2.zip › DF1/PXD010154/Colon/Colon_10_MAPKAPK5_QVIEEQTTSHESTGGAK.pdf]

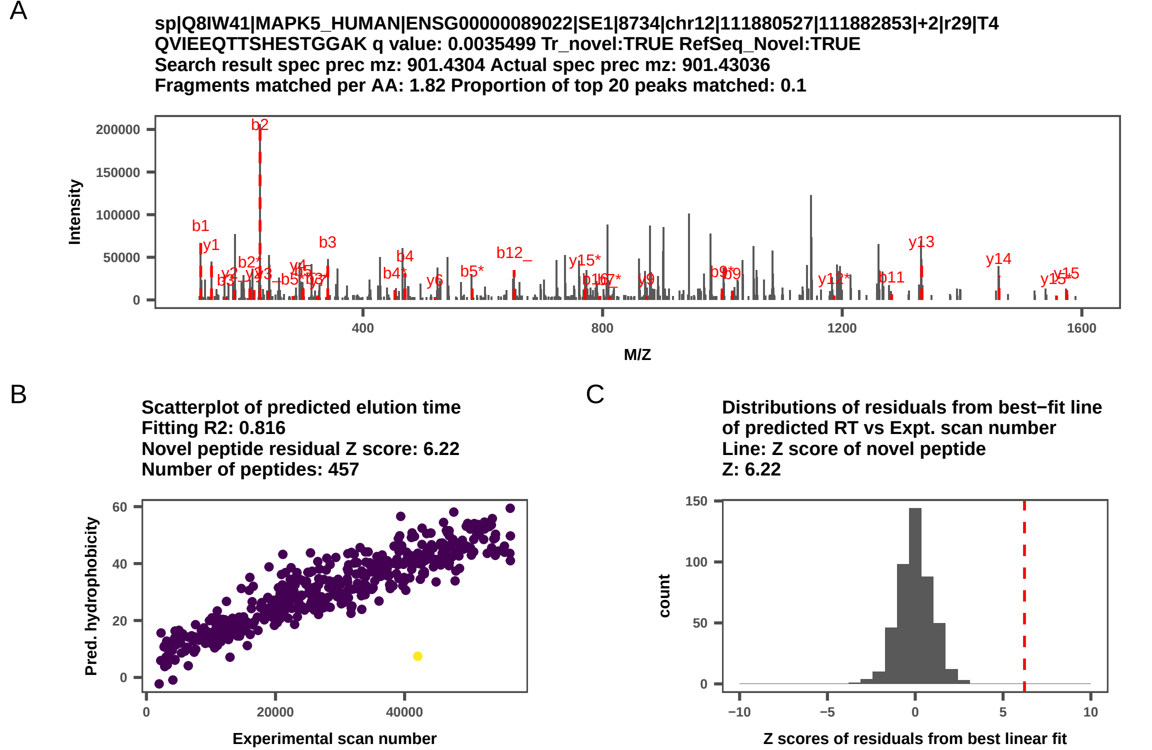
A
sp|Q8IW41|MAPK5_HUMAN|ENSG00000089022|SE1|8734|chr12|111880527|111882853|+2|r29|T4
QVIEEQTTSHESTGGAK q value: 0.0035499 Tr_novel:TRUE RefSeq_Novel:TRUE
Search result spec prec mz: 901.4304 Actual spec prec mz: 901.43036
Fragments matched per AA: 1.82 Proportion of top 20 peaks matched: 0.1
Intensity
200000
150000
100000
50000
0
400
800
1200
1600
M/Z
b1
y1
b2
b3
b4
y6
b5*
b12_
y15*
y9
b9
y12*
b11
y13
y14
B
Scatterplot of predicted elution time
Fitting R2: 0.816
Novel peptide residual Z score: 6.22
Number of peptides: 457
Pred. hydrophobicity
60
40
20
0
20000
40000
Experimental scan number
C
Distributions of residuals from best−fit line
of predicted RT vs Expt. scan number
Line: Z score of novel peptide
Z: 6.22
count
150
100
50
0
−10
−5
5
10
Z scores of residuals from best linear fit
